# Profiles of HBcrAg and pgRNA in Pregnant Women With Chronic HBV Under Different Disease Phases and Antiviral Prophylaxis

**DOI:** 10.1093/ofid/ofae241

**Published:** 2024-05-03

**Authors:** Chun-Rui Wang, Xiao-qin Liu, Wei Shen, Guo-Chao Zhong, Hu Li, Qiao Tang, Yu-Xing Liu, Peng Hu

**Affiliations:** Department of Infectious Diseases, Institute for Viral Hepatitis, The Key Laboratory of Molecular Biology for Infectious Diseases, Chinese Ministry of Education, The Second Affiliated Hospital of Chongqing Medical University, Yuzhong District, Chongqing, China; Department of Infectious Diseases, Institute for Viral Hepatitis, The Key Laboratory of Molecular Biology for Infectious Diseases, Chinese Ministry of Education, The Second Affiliated Hospital of Chongqing Medical University, Yuzhong District, Chongqing, China; Department of Infectious Diseases, Institute for Viral Hepatitis, The Key Laboratory of Molecular Biology for Infectious Diseases, Chinese Ministry of Education, The Second Affiliated Hospital of Chongqing Medical University, Yuzhong District, Chongqing, China; Department of Hepatobiliary Surgery, The Second Affiliated Hospital of Chongqing Medical University, Yuzhong District, Chongqing, China; Department of Infectious Diseases, Institute for Viral Hepatitis, The Key Laboratory of Molecular Biology for Infectious Diseases, Chinese Ministry of Education, The Second Affiliated Hospital of Chongqing Medical University, Yuzhong District, Chongqing, China; Department of Infectious Diseases, Institute for Viral Hepatitis, The Key Laboratory of Molecular Biology for Infectious Diseases, Chinese Ministry of Education, The Second Affiliated Hospital of Chongqing Medical University, Yuzhong District, Chongqing, China; Department of Infectious Diseases, Institute for Viral Hepatitis, The Key Laboratory of Molecular Biology for Infectious Diseases, Chinese Ministry of Education, The Second Affiliated Hospital of Chongqing Medical University, Yuzhong District, Chongqing, China; Department of Infectious Diseases, Institute for Viral Hepatitis, The Key Laboratory of Molecular Biology for Infectious Diseases, Chinese Ministry of Education, The Second Affiliated Hospital of Chongqing Medical University, Yuzhong District, Chongqing, China

**Keywords:** different stages, HBcrAg, HBV in pregnancy, mother-to-child transmission, pgRNA

## Abstract

**Background:**

Pregnant women with chronic hepatitis B (CHB) exhibit unique clinical features in terms of postpartum immune system reconstitution and recovery from pregnancy-related changes. However, current studies focus primarily on the outcomes of maternal–infant transmission and postpartum hepatitis flares. We aimed to evaluate the profiles of hepatitis B core-related antigen (HBcrAg) and pregenomic RNA (pgRNA) in pregnant women with CHB.

**Methods:**

This retrospective analysis included treatment-naïve pregnant women with CHB who were followed up regularly in an outpatient clinic from 2014 to 2021. Baseline HBcrAg and pgRNA levels were compared in patients with different disease phases. Changes in these parameters were examined in a subset of patients receiving antiviral prophylaxis. HBcrAg and pgRNA levels were measured before treatment, at 32 weeks of gestation, and postpartum.

**Results:**

The final analysis included a total of 121 patients, 100 of whom were hepatitis B e antigen (HBeAg)–positive (96 and 4 in the immune-tolerant and -indeterminate phases, respectively) and 21 of whom were HBeAg-negative (6 and 15 in the immune-active and -inactive carrier phases, respectively). The HBeAg-negative group vs the HBeAg-positive group had lower levels of baseline HBcrAg (median [interquartile range {IQR}], 3.7 [3.0–5.9] vs 8.6 [8.4–8.7] log_10_ U/mL; *P* < .01) and pgRNA (median [IQR], 0.0 [0.0–2.5] vs 7.8 [7.6–8.1] log_10_ copies/mL; *P* < .01). The serum levels of HBcrAg and pgRNA were highest in immune-tolerant carriers and lowest in immune-inactive carriers. In HBeAg-positive patients, the correlation coefficients of HBcrAg and pgRNA with hepatitis B virus (HBV) DNA were 0.40 and 0.43, respectively; in HBeAg-negative patients, they were 0.53 and 0.51, respectively (all *P* < .05). The correlation coefficients with hepatitis B surface antigen (HBsAg) were 0.55 and 0.52 (*P* < .05) in HBeAg-positive patients, respectively, while in HBeAg-negative patients they were 0.42 and 0.37, respectively (*P* > .05). Among 96 patients receiving antiviral prophylaxis, we detected a rapid decrease in HBV DNA to an undetectable level during treatment but relatively stable levels of pgRNA and HBcrAg.

**Conclusions:**

HBcrAg and pgRNA levels are lower in HBeAg-negative patients than in HBeAg-positive patients. These 2 markers are significantly associated with HBV DNA irrespective of HBeAg status, while they are significantly associated with HBsAg only in HBeAg-positive patients.

Chronic hepatitis B virus (HBV) infection is a dynamic disease characterized by complex interactions between HBV, hepatocytes, and the immune system of the host. Currently, serum viral markers, including hepatitis B surface antigen (HBsAg), hepatitis B e antigen (HBeAg), HBV DNA, and alanine aminotransferase (ALT), are widely used to define distinct phases of chronic HBV infection [1]. However, these markers are not sufficient to recognize patients in gray zones or indeterminate phases [2]. Serum pregenomic RNA (pgRNA) and hepatitis B core-related antigen (HBcrAg), 2 promising surrogate markers of cccDNA, are recognized as potential novel markers for predicting treatment response and prognosis of chronic hepatitis B (CHB) patients [3]. Serum pgRNA is an intermediate in the process of HBV replication and is suggested to be related to the risk of viral rebound after treatment cessation [4]. In addition, serum HBV RNA levels also reflect intrahepatic viral transcriptional activity during nucleos(t)ide analog therapy [5] and are related to HBeAg status in the HBV-infected population. HBcrAg, a complex of viral proteins [6], is helpful for identifying different phases of chronic HBV infection, especially in HBeAg-negative patients [7], and could be a potential marker for disease monitoring and disease outcomes in CHB patients [6]. These 2 novel markers may have the potential to be utilized in characterizing the natural progression of chronic HBV infection.

Notably, current studies on pgRNA and HBcrAg are almost always performed in HBV-infected nonpregnant patients. Chronic HBV infection during pregnancy presents unique and challenging issues. Maternal cell-mediated immunity is suppressed to maintain immunological tolerance to avoid rejection of the fetus during pregnancy. This suppression is reversed postpartum, which could influence the course or natural history of HBV infection [8]. Currently, only 3 reports have described the clinical utility of pgRNA or HBcrAg among pregnant women with chronic HBV [9–11]; however, they did not describe pgRNA or HBcrAg kinetics under different disease phases and antiviral therapies among this special population. Our previous study revealed that pgRNA was not only positively correlated with classic viral biomarkers but also acted as an independent predictor of HBsAg reduction postpartum among pregnant HBeAg-positive carriers with chronic HBV [12]. Hence, we hypothesized that these 2 biomarkers could also reflect the profiles of maternal virology levels in pregnant women with HBV infection. We aimed to investigate the roles of HBcrAg and pgRNA and their correlations with other markers in pregnant women with chronic HBV with or without treatment.

## METHODS

### Study Population

Pregnant women were enrolled from the Second Affiliated Hospital of Chongqing Medical University outpatient clinic between January 2019 and September 2021. Pregnant women with chronic HBV were included, regardless of whether they received treatment during pregnancy. The following patients were excluded: (1) those who received treatment before pregnancy; (2) those with autoimmune hepatitis or pregnancy-related diseases; (3) those who were coinfected with hepatitis A/C/D/E or HIV; or (4) those with severe hepatitis, fibrosis, or cirrhosis (severe hepatitis was defined as ALT ≥5 upper limit of normal [40 U/L]) associated with coagulopathy (prothrombin time <50% and/or INR >1.5). Severe fibrosis or cirrhosis was defined as a FibroScan score >12.0 kPa, taking into account the liver disease etiology, or a Metavir score of ≥F3 [13, 14]. All patients underwent postpartum follow-up evaluation for ≥8 weeks under an approved ethics protocol from the Second Affiliated Hospital of Chongqing Medical University. Written informed consent was obtained from all patients. This study was registered with the Chinese Clinical Trial Registry (ChiCTR2100054116). The flowchart for identifying eligible patients is shown in Supplementary Figure 1. Included patients were categorized into 4 subgroups based on different disease phases: immune tolerant in HBeAg-positive patients (IT(e)), indeterminate in HBeAg-positive patients (IND(e)), immune active in HBeAg-negative patients (IA(e−), and inactive carrier phase in HBeAg-negative patients (IC(e−)) [1]. For patients on treatment, serum samples were collected at 24–28 weeks of gestation before treatment (referred to as baseline), 32–36 weeks of gestation (referred to as near delivery), and 2–6 weeks after delivery (referred to as the postpartum period). Flowcharts showing identification of the study population and the time points at which samples were collected are shown in Supplementary Figures 1 and 2, respectively.

### Clinical and Laboratory Assessments

Clinical and laboratory assessments were performed every 4 or 12 weeks during pregnancy and postpartum. HBV DNA levels and serological marker levels were measured using a real-time polymerase chain reaction kit (Kehua Bioengineering Co., Ltd., Shanghai, China) and an enzyme-linked immunosorbent assay kit (Abbott, Chicago, IL, USA), respectively. Serum ALT levels were assessed at central laboratories as per standard procedures. Serum HBcrAg was quantified by a LUMIPULSE G1200 chemiluminescent immunoassay, with a sensitivity of 2 log U/mL. Serum pgRNA quantification was assessed by an ABI7500 quantitative real-time polymerase chain reaction system, with a detection range from 2 × 10^2^ to 1 × 10^9 ^copies/mL. If the index value was lower than the sensitivity, the results were considered “detectable but not quantifiable (UQ).” To assess the reverse transcriptional efficiency of pgRNA in the various chronic HBV infection phases, the ratio of serum HBV RNA to HBV DNA was calculated. The aspartate aminotransferase-to-platelet ratio (APRI) and fibrosis 4 score (Fib-4) were calculated as previously described [15].

### Statistical Analysis

All the data analyses were conducted with SPSS software, version 26.0 (SPSS Inc.), and R 3.6.1 software. Categorical and continuous variables are expressed as counts (percentages) and medians (ranges), respectively. The chi-square test or Fisher exact test was used to compare categorical variables. The longitudinal data were visualized through scatter plots over time and analyzed using linear regression models (with robust standard errors to accommodate the repeated measurements). Additionally, box plots were generated for presentation, and paired tests were utilized for analysis. Spearman's correlation test was performed to analyze the correlations of pgRNA and HBcrAg levels with other classic serum markers. Serum HBsAg, HBV DNA, pgRNA, and HBcrAg levels are expressed as log values. A 2-sided *P* < .05 was considered significant for all the statistical tests.

## RESULTS

### Baseline Characteristics of Patients

A total of 152 pregnant women with HBV infection were invited to participate in our study from January 2014 to September 2021. Finally, 121 patients were included, and 31 were excluded due to prior receipt of HBV therapy. The median age (interquartile range [IQR]) of these patients was 28.0 (26.0–31.0) years. Patient characteristics are summarized in Table 1. There were 100 HBeAg-positive patients and 21 HBeAg-negative patients. Compared with the HBeAg-negative patients, the HBeAg-positive patients had higher levels of HBV DNA (median [IQR], 6.9 [6.5–7.4] vs 3.1 [2.0–4.7] log_10_ IU/mL), pgRNA (median [IQR], 7.8 [7.6–8.1] vs 0.0 [0.0–2.5] log_10_ copies/mL), and HBcrAg (median [IQR], 8.6 [8.4–8.7] vs 3.7 [3.0–5.9] log_10_ U/mL). There were no significant differences in age, ALT level, APRI, or FIB4 between the 2 groups (all *P* > .05). The distribution of pgRNA and HBcrAg levels according to HbeAg status is shown in Supplementary Figure 3.

**Table 1.** Clinical Characteristics of 121 Enrolled Patients at Baseline

Parameter	Whole Cohort	HBeAg-Positive Group	HBeAg-Negative Group	*P* Value
	n = 121	n = 100	n = 21	
Age, y	28.0 (26.0–31.0)	28.0 (26.0–31.0)	29.0 (28.0–31.0)	.126
Parity status, No. (%)				.048
The first pregnancy	111 (91.7)	94 (94.0)	17 (81.0)	
The second pregnancy	10 (8.3)	6 (6.0)	4 (19.0)	
Infant's sex, male, No. (%)	56 (46.3)	44 (44.0)	12 (57.1)	.272
ALT, U/L	22.0 (16.0–33.0)	23.5 (16.0–33.0)	21.0 (14.0–30.0)	.294
ALT categories, No. (%)				.224
≤19 U/L	50 (41.32)	41 (41.00)	9 (42.86)	
>19–40 U/L	55 (45.45)	48 (48.00)	7 (33.33)	
>40 U/L	16 (13.22)	11 (11.00)	5 (23.81)	
Positive detection rate, No. (%)^a^				
pgRNA	105 (86.78)	98 (98.00)	7 (33.33)	<.001
HBV DNA	114 (94.21)	98 (98.00)	16 (76.19)	<.001
HBsAg	121 (100.00)	100 (100.00)	21 (100.00)	.374
HBcrAg	118 (97.52)	100 (100.00)	18 (85.71)	<.005
pgRNA, log_10_ copies/mL	7.7 (6.5–8.0)	7.8 (7.6–8.1)	0.0 (0.0–2.5)	<.001
HBV DNA, log_10_ IU/mL	6.7 (5.6–7.3)	6.9 (6.5–7.4)	3.1 (2.0–4.7)	<.001
pgRNA/HBV DNA ratio, log_10_ copies/mL/IU/mL	0.8 (0.0–1.2)	0.9 (0.4–1.2)	−1.1 (−3.0–0.0)	<.001
HBsAg, log_10_ IU/mL	4.4 (3.7–4.6)	4.5 (4.2–4.7)	3.2 (2.7–3.6)	<.001
HBcrAg, log_10_ U/mL	8.4 (7.7–8.7)	8.6 (8.4–8.7)	3.7 (3.0–5.9)	<.001
HBeAg, log_10_ PEIU/mL	3.1 (2.0–3.3)	3.2 (2.9–3.3)	NA	
APRI	0.3 (0.3–0.5)	0.3 (0.3–0.5)	0.3 (0.2–0.4)	.065
APRI categories, No. (%)				.080
≤1	117 (96.69)	98 (98.00)	19 (90.48)	
>1	4 (3.31)	2 (2.00)	2 (9.52)	
FIB-4	0.8 (0.6–1.0)	0.8 (0.6–1.0)	0.7 (0.6–1.1)	.235
FIB-4 categories, No. (%)				.120
≤1.45	110 (90.91)	92 (92.00)	18 (85.71)	
>1.45	8 (6.61)	5 (5.00)	3 (14.29)	

Continuous variables are expressed as median (IQR), and categorical variables are expressed as count (percentage).

Abbreviations: ALT, alanine aminotransferase; APRI, Aspartate aminotransferase-to-Platelet Ratio Index; FIB-4, Fibrosis 4 score; HBcrAg, hepatitis B core-related antigen; HBeAg, hepatitis B e antigen; HBsAg, hepatitis B surface antigen; HBV, hepatitis B virus; IQR, interquartile range; LDT, telbivudine; MTCT, mother-to-child transmission; NA, not available; pgRNA, pregenomic RNA; TDF, tenofovir disoproxil fumarate.

^a^Positive detection rate of pgRNA, HBV DNA, HBsAg, and HBcrAg would refer to the proportion of individuals in a group who test positive for the presence of pgRNA, HBV DNA, HBsAg, and HBcrAg, respectively.

Among the HbeAg-positive patients, 96 (96.0%) and 4 (4.0%) were in the immune-tolerant (IT(e+)) and immune-indeterminant (IND(e+)) phases, respectively. Among the HBeAg-negative patients, 6 (28.6%) and 15 (71.4%) individuals were in the immune-active (IA(e−)) and inactive-carrier (IC(e−)) phases, respectively. The baseline characteristics of 121 pregnant patients across different CHB phases are summarized in Supplementary Table 1. Patients in the IT(e+) phase had higher levels of all viral markers (HBV DNA, HBeAg, HBsAg, pgRNA, and HBcrAg) than did those in the IND(e+) phase, while patients in the IA(e−) phase had higher levels of only HBV DNA and HBcrAg than did those in the IC(e−) phase. All viral markers were positive in all patients in the IT(e+) phase. The percentage of pgRNA-positive patients decreased by half in the IND(e+) and IA(e−) phases, further decreasing to 26.67% in the IC(e−) phase, while the percentage of serum HBcrAg-positive patients in the IND(e+) and IA(e−) phases decreased to 80.00% in the IC(e−) phase.

### Profiles of HBcrAg and pgRNA in Different Disease Phases

**Figure 1.**
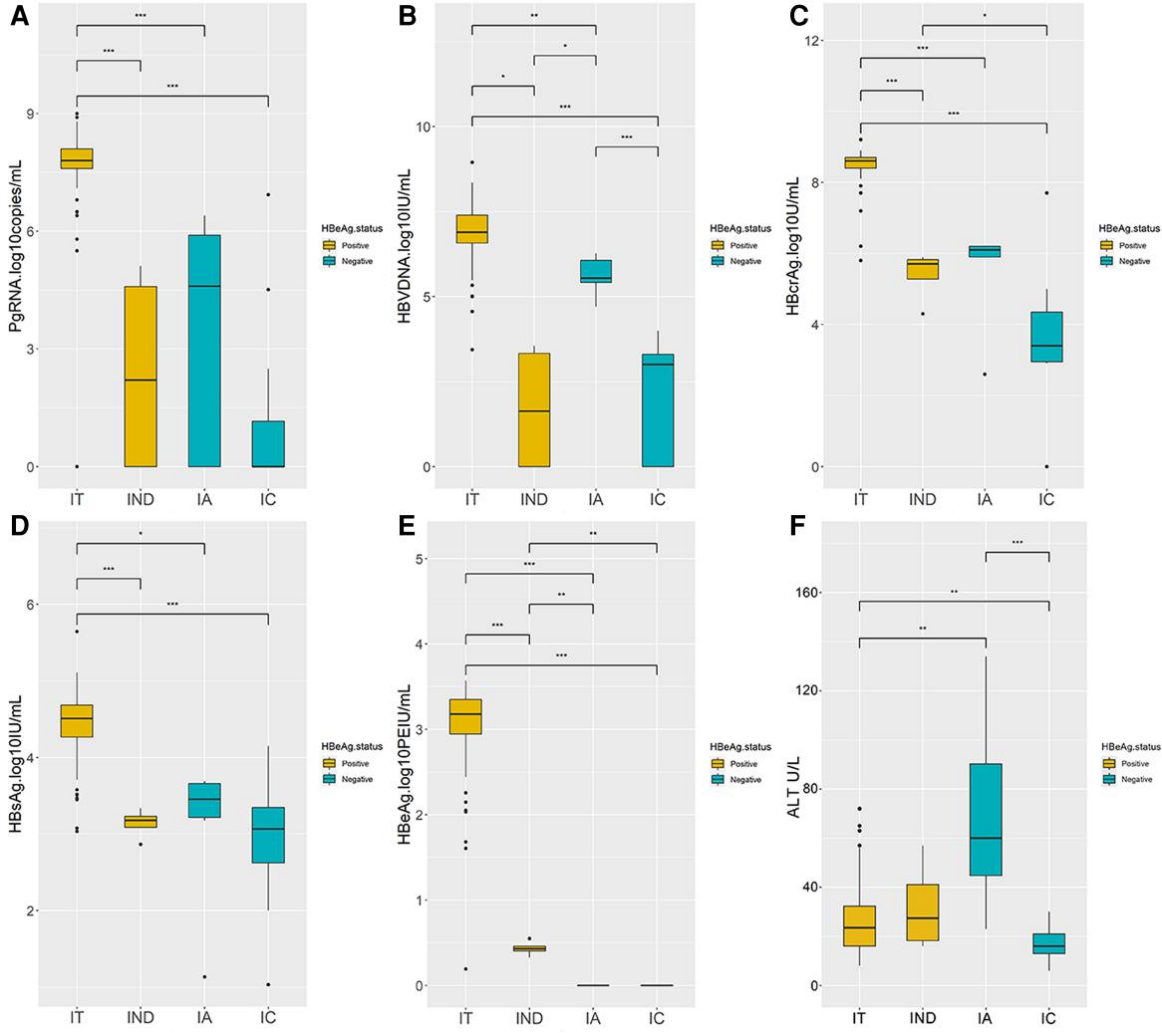
Profile of HBV viral markers (*A*–*E*) and ALT levels (*F*) in different disease phases of CHB infection with pregnancy at baseline. Abbreviations: ALT, alanine aminotransferase; CHB, chronic hepatitis B; HBV, hepatitis B virus.

At baseline, the median levels of all viral markers were highest in patients in the IT(e+) phase and lowest in those in the IC(e−) phase (Figure 1). The median pgRNA/HBV DNA ratio was ∼1 in the IT(e+) phase and <1 in the other phases. The pgRNA/HBV DNA ratio was significantly greater in HBeAg-positive patients than in HBeAg-negative patients (0.90 vs −1.10; *P* < .001), while it was not significantly different for HBeAg-positive patients in the IT and IND phases (0.90 vs 0.40; *P* = .61) or HBeAg-negative patients in the IA and IC phases (−2.9 vs −1.1; *P* = .15) (Tables 1; Supplementary Table 1).

**Figure 2.**
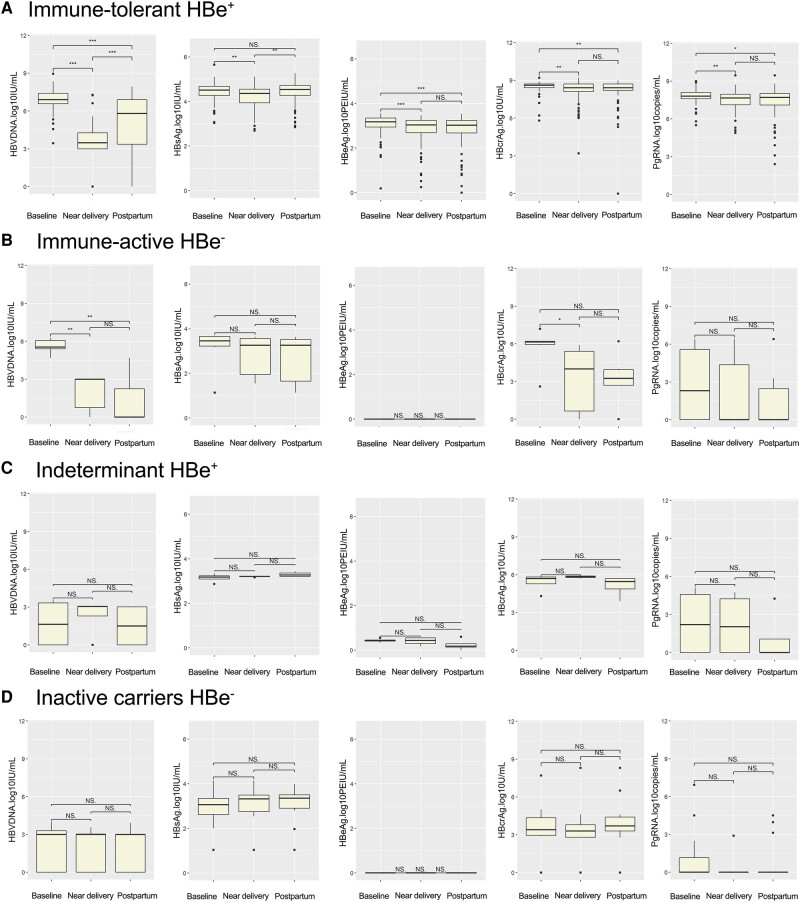
Kinetics of HBV viral markers from baseline to postpartum in 4 groups. Kinetics of HBV DNA, HBsAg, HBeAg, HBcrAg, and pgRNA levels are shown in IT(e+) phase (*A*), IA(e−) (*B*), IND(e+) phase (*C*), and IC(e−) phase (*D*). Baseline, 24–28 weeks of gestation; near delivery, 32–36 weeks of gestation; postpartum, 4–8 weeks after delivery. Nonsignificant *P* > .05. *P < .05; **P < .01; ***P < .001. Abbreviations: HBcrAg, hepatitis B core-related antigen; HBeAg, hepatitis B e antigen; HBsAg, hepatitis B surface antigen; HBV, hepatitis B virus; IA, immune active; IC, inactive; IND, indeterminate; IT, immune tolerant; NS, nonsignificant; pgRNA, pregenomic RNA.

**Figure 3.**
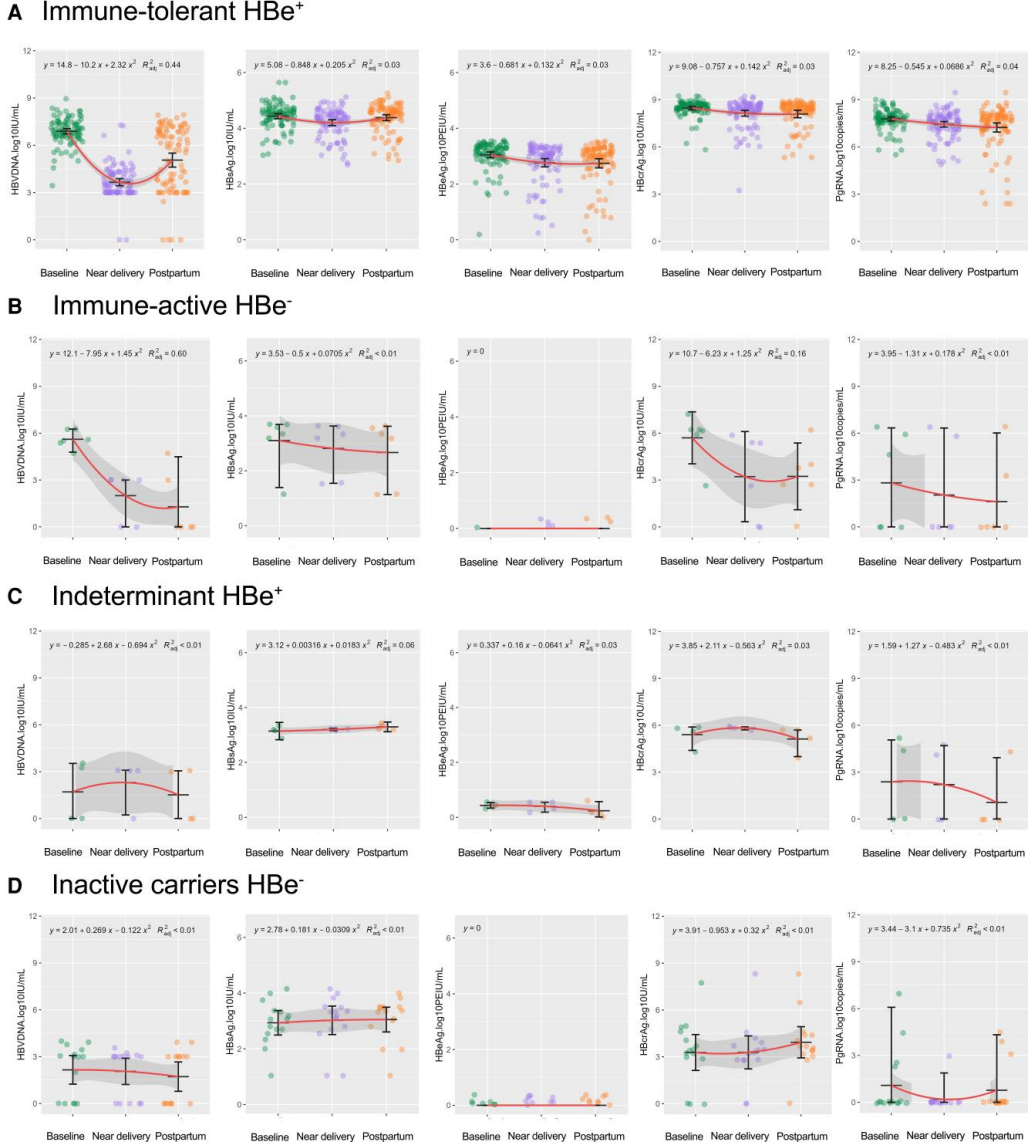
Linear regression models and associated 95% CIs were plotted over scatter plots to descriptively assess the profile of HBV viral markers from baseline to postpartum in 4 groups. Scatter plots of HBV DNA, HBsAg, HBeAg, HBcrAg, and pgRNA levels are shown in IT(e+) phase (*A*), IA(e−) (*B*), IND(e+) phase (*C*), and IC(e−) phase (*D*). Baseline, 24–28 weeks of gestation; near delivery, 32–36 weeks of gestation; postpartum, 4–8 weeks after delivery. Abbreviations: HBcrAg, hepatitis B core-related antigen; HBeAg, hepatitis B e antigen; HBsAg, hepatitis B surface antigen; HBV, hepatitis B virus; IA, immune active; IC, inactive; IND, indeterminate; IT, immune tolerant; pgRNA, pregenomic RNA.

In the IT(e+) phase, all patients received antiviral prophylaxis at 24–28 weeks of gestation [15]. We analyzed the kinetics of pgRNA and HBcrAg levels during pregnancy and postpartum. Compared with those at baseline, the serum pgRNA, HBcrAg, and HBV DNA levels decreased near delivery and postpartum, while the HBsAg level remained stable during the postpartum period (Figures 2*A* and 3*A*). Viral markers were positive in all patients except for HBV DNA, which was positive in 64.58% of patients near delivery. At 2–6 weeks postpartum, 58.97% and 94.87% of patients were positive for serum HBV DNA and HBeAg, respectively, while all patients were positive for other viral markers.

A different phenomenon was observed among the 6 patients in the IA(e−) phase. These 6 patients received antiviral treatment for hepatitis [15]. Only the serum HBV DNA concentration decreased from baseline to postpartum, and the serum HBcrAg concentration decreased near delivery compared with that at baseline. Other viral markers remained unchanged during pregnancy and postpartum (Figures 2*B* and 3*B*). At delivery, 33.33%, 0%, 100%, and 66.67% of the patients were positive for serum pgRNA, HBV DNA, HBsAg, and HBcrAg, respectively. After delivery, 1 patient treated with LDT was lost to follow-up. The above 4 biomarkers were positive in 20%, 0%, 100%, and 80% of patients, respectively.

Another 19 patients were not treated for their lower viral load at baseline (median [IQR] of HBV DNA, 3.0 [0–3.4] log_10_ IU/mL), which included 4 patients in the IND(e+) phase and 15 patients in the IC(e−) phase. During follow-up, all viral markers remained stable from baseline to postpartum (Figure 2*C*, *D* and 3*C*, *D*).

### Associations Between Serum pgRNA and HBcrAg Levels and Other Serum Marker Levels at Baseline

**Figure 4.**
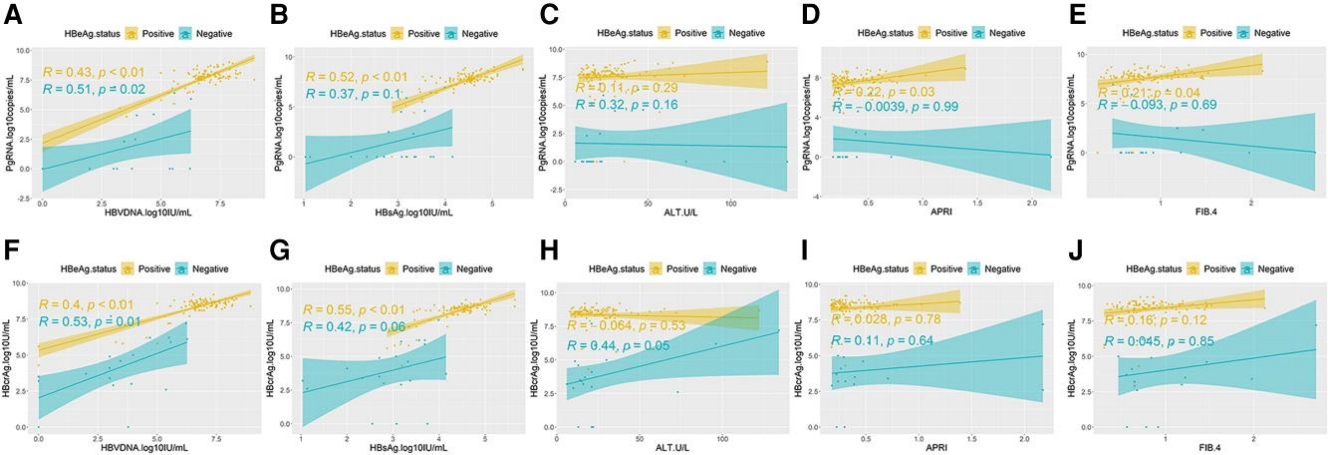
Association between pgRNA (*A*–*E*) and HBcrAg (*F*–*J*) with other markers at baseline. R = Spearman’s correlation coefficient; *P* = *P* value of correlation *t* test. Abbreviations: HBcrAg, hepatitis B core-related antigen; pgRNA, pregenomic RNA.

pgRNA levels correlated significantly with HBV DNA levels independent of HBeAg status (e+ R = 0.43; *P* < .01; e– R = 0.51; *P* < .02) (Figure 4*A*) and with HBsAg (e+ R = 0.52; *P* < .01) (Figure 4*B*) and HBeAg (e+ R = 0.62; *P* < .01) among HBeAg-positive patients. The correlation between pgRNA and HBsAg was not significant among HBeAg-negative patients (e– R = 0.37; *P* = .1) (Figure 4*B*). We also described the associations between pgRNA levels and liver disease markers (Figure 4*C*–*E*). Among HBeAg-positive patients, there were weak positive associations between pgRNA levels and APRI (*P* = .03) and FIB-4 (*P* = .04), while the association with ALT (*P* = .29) was not significant. Serum pgRNA levels were not associated with ALT (*P* = .16), APRI (*P* = .99), or FIB-4 (*P* = .69) among HBeAg-negative patients.

Similarly, HBcrAg levels correlated weakly with HBV DNA levels independent of HBeAg status (e+ R = 0.40; *P* < .01; e– R = 0.53; *P* = .01) (Figure 4*F*) and moderately with HBsAg among HBeAg-positive patients (e+ R = 0.55; *P* < .01) (Figure 4*G*) and HBeAg (e+ R = 0.75; *P* < .01). The correlation between HBcrAg and HBsAg was not significant among HBeAg-negative patients (e– R = 0.42; *P* = .06) (Figure 4*G*). We also described the associations between HBcrAg levels and liver disease markers (Figure 4*H*–*J*). Among HBeAg-positive patients, HBcrAg levels were not associated with ALT (*P* = .53), APRI (*P* = .78), or FIB-4 (*P* = .12). Among HBeAg-negative patients, there was a weak positive association between HBcrAg levels and ALT (*P* = .05), while associations with APRI (*P* = .64) and FIB-4 (*P* = .85) were not significant.

### Associations Among Viral Markers During Antiviral Prophylaxis

Until 2–6 weeks postpartum, only 39 serum samples were collected. The correlation coefficients between the viral biomarkers are shown in Table 2. The levels of pgRNA were correlated with the levels of HBV DNA, HBcrAg, HBsAg, and HBeAg before treatment (R = 0.353, 0.523, 0.456, and 0.259, respectively; all *P* < .05). The association between pgRNA and HBV DNA was still preserved near delivery and postpartum (R = 0.300 and 0.332, respectively; both *P* < .05) but strengthened between pgRNA and HBcrAg, HBsAg, and HBeAg near delivery (R = 0.652, 0.581, and 0.471, respectively; all *P* < .001) and postpartum (R = 0.799, 0.810, and 0.636; all *P* < .001).

**Table 2.** Correlation Coefficients Between Different Viral Biomarkers in 96 Patients Receiving Antiviral Prophylaxis

	pgRNA, log_10_ copies/mL	HBcrAg, log_10_ U/mL	HBsAg, log_10_ IU/mL	HBeAg, log_10_ PEIU/mL
Baseline (n = 96)				
HBV DNA, log_10_ IU/mL	0.353 (*P* < .000)	0.324 (*P* = .001)	0.314 (*P* = .002)	0.207 (*P* = .043)
pgRNA, log_10_ copies/mL	…	0.523 (*P* < .000)	0.456 (*P* < .000)	0.259 (*P* = .011)
HBcrAg, log_10_ U/mL	…	…	0.490 (*P* < .000)	0.513 (*P* < .000)
HBsAg, log_10_ IU/mL	…	…	…	0.538 (*P* < .000)
Near delivery (n = 96)				
HBV DNA, log_10_ IU/mL	0.300 (*P* = .003)	0.182 (*P* = .076)	0.374 (*P* < .000)	0.263 (*P* = .010)
pgRNA, log_10_ copies/mL	…	0.652 (*P* < .000)	0.581 (*P* < .000)	0.471 (*P* < .000)
HBcrAg, log_10_ U/mL	…	…	0.475 (*P* < .000)	0.660 (*P* < .000)
HBsAg, log_10_ IU/mL	…	…	…	0.583 (*P* < .000)
Postpartum (n = 39)				
HBV DNA, log_10_ IU/mL	0.332 (*P* = .039)	0.173 (*P* = .291)	0.332 (*P* = .039)	0.332 (*P* = .039)
pgRNA, log_10_ copies/mL	…	0.799 (*P* < .000)	0.810 (*P* < .000)	0.636 (*P* < .000)
HBcrAg, log_10_ U/mL	…	…	0.627 (*P* < .000)	0.739 (*P* < .000)
HBsAg, log_10_ IU/mL	…	…	…	0.627 (*P* < .000)

Correlation coefficients were calculated by Spearman's test; *P* = *P* value of correlation *t* test.

Abbreviations: HBcrAg, hepatitis B core-related antigen; HBeAg, hepatitis B e antigen; HBsAg, hepatitis B surface antigen; HBV, hepatitis B virus; pgRNA, pregenomic RNA.

HBcrAg also showed significant correlations with HBV DNA, HBsAg, and HBeAg at baseline (R = 0.324, 0.490, and 0.513, respectively; all *P* < .05). The association between HBcrAg and HBsAg was still preserved near delivery (R = 0.475; *P* < .001) and strengthened postpartum (R = 0.627; *P* < .001) but weakened between HBcrAg and HBV DNA near delivery and postpartum (R = 0.182 and 0.173, respectively; both *P* > .05).

## DISCUSSION

In this study of pregnant participants with CHB in Southwest China, we described serum pgRNA and HBcrAg kinetics and investigated their associations with classic viral and clinical markers during pregnancy and the postpartum period. At baseline, serum pgRNA and HBcrAg levels were lower in HBeAg-negative patients than in HBeAg-positive patients, in line with previous findings in smaller patient cohorts [16, 17], which could be related to the diminished production of HBeAg after HBeAg seroconversion. Canadian investigators [18] identified HBeAg status as an independent factor associated with serum HBV RNA levels, which probably explained the greater transcriptional activity of cccDNA in HBeAg-positive patients than in HBeAg-negative patients. We calculated the serum pgRNA/DNA ratio to reflect the reverse transcriptional efficiency of the pgRNA. The results showed that it was greater in HBeAg-positive patients than in HBeAg-negative patients, which is consistent with the findings of prior studies [19, 20], but the ratios were similar between the IT(e+) and IND(e+) phases and between the IA(e−) and IC(e−) phases, in contrast to a recently published study showing a significantly greater ratio in the HBeAg-negative chronic hepatitis group than in the HBeAg-negative chronic infection group (1.04 vs 0.85; *P* < .01) [20]. This discrepancy could be related to the definition of the group and the lower limit of detection of pgRNA and HBV DNA. In addition, we found that the median pgRNA/HBV DNA ratio was <1 in HBeAg-negative patients. The reason was uncertain and could be related to the increased probability of the detection of integrated HBV DNA in HBeAg-negative participants [21].

To date, a number of studies have investigated the clinical implications of pgRNA or HBcrAg in nucleos(t)ide analog (NA)-treated CHB patients [22]. Nonetheless, only 3 studies have analyzed the dynamic changes in serum pgRNA or HBcrAg among pregnant women with chronic HBV receiving long-term NA treatment [9–11]. An analysis of 96 patients receiving antiviral prophylaxis revealed a rapid decrease in HBV DNA to an undetectable level but relatively stable levels of serum pgRNA and HBcrAg. Mechanistically, NAs can block the reverse transcription of encapsidated pgRNA to DNA but have no direct effect on cccDNA or its transcription, resulting in short-term pgRNA accumulation and release into the circulation, which has been confirmed in prior studies [10, 19]. Although patients treated with NA often have undetectable serum HBV DNA, most of them still have detectable cccDNA, which could explain why most pregnant patients infected with HBV experienced virological relapse after treatment cessation postpartum. This phenomenon was confirmed in our previously published article [23]. These findings support the conclusions of other studies that serum pgRNA or HBcrAg levels are useful for monitoring antiviral effects.

Consistent with previous studies, we concluded that there were stable associations between pgRNA, as well as between HBcrAg and HBV DNA levels, among both HBeAg-positive and HBeAg-negative patients [16, 17, 19, 20]. Interestingly, we found that both serum pgRNA (R = 0.67; *P* < .001) and HBcrAg (R = 0.57; *P* < .001) correlated well with serum HBsAg in HBeAg-positive participants, but there was no significant correlation between serum pgRNA and HBsAg (R = 0.13; *P* = .37) or between serum HBcrAg and HBsAg (R = 0.24; *P* = .05) in HBeAg-negative participants. This discrepancy supported the emerging concept that the source of circulating HBsAg in many HBeAg-negative patients may be derived from HBV DNA fragments integrated into the human genome, not cccDNA [24]. Serum HBsAg can also be derived from the expression of the integrated HBV S gene in patients with S gene integration [25]. Thus, to a certain extent, serum pgRNA or HBcrAg may be more stable than serum HBsAg in reflecting intrahepatic cccDNA. We did not find a significant or strong correlation between these 2 novel markers and clinical markers in the present study, while another study reported that both HBV RNA and HBcrAg levels were consistently positively associated with liver disease markers (ALT, APRI, and FIB-4) among HBeAg-negative patients [26]. The participants in the 2 studies were different, and pregnant women presented unique immune characteristics, which could explain the different conclusions of the 2 studies. However, more studies are needed to confirm the correlation between pgRNA or HBcrAg and liver disease markers.

In addition, we found that serum pgRNA level correlated well with HBcrAg level during follow-up among patients receiving antiviral prophylaxis (R = 0.523–0.799), which is consistent with the findings of a previous study reporting a strong positive correlation between pgRNA and HBcrAg in patients receiving nucleos(t)ide analog therapy [27]. Both of these genes originate from cccDNA, which may explain the relatively strong correlation between serum pgRNA and HBcrAg. Serum pgRNA and HBcrAg could become targets for novel antiviral therapy in the future [28], such as RNA interfering gene silencers targeting HBV mRNA translation and capsid assembly inhibitors destabilizing core protein assembly and inhibiting capsid formation. Ram W. Sabnis [29] described a combination therapy of RNA interference agents and small molecules of capsid assembly modulators that were useful for the treatment of HBV infection. Patients achieved robust reductions in HBV RNA but fewer reductions in HBcrAg. Although pgRNA and HBcrAg showed similar kinetics and correlated well with each other during nucleos(t)ide analog therapy, their individual roles in research on novel antiviral agents were not completely consistent [30]. A phase 2b trial [31] in which JNJ-3989 was combined with NAs reported that the reductions in HBsAg and HBV RNA were more pronounced than those in HBeAg and HBcrAg. Another trial of short-term RNA interference showed suppressive effects on HBV RNA and HBsAg reduction, with less pronounced effects on HBcrAg [32]. However, further studies are needed to determine the role of pgRNA and HBcrAg in patients receiving novel agent treatment.

To our knowledge, this is the first study to report the profiles and correlation of 2 novel HBV biomarkers (serum pgRNA and HBcrAg) in pregnant women with chronic HBV on oral nucleos(t)ide analog therapy in China. There are several limitations in our research. Our study is based on real-life clinical retrospective practice with inevitable retrospective bias. First, the sample size and number of HBeAg-negative patients were relatively small. Additional larger, prospective cohorts are needed to confirm the present findings. Second, there are various commercial testing methods for serum HBV RNA quantification; thus, standardized testing methods and agents are urgently needed. Third, the HBV genotype was not detected. We described the phenotype without any hypotheses, and further experiments are needed to determine the reason for this phenomenon. The major HBV genotypes in China are B and C, while in Europe A and D are the main genotypes. Therefore, genotypes should also be considered in future studies to obtain comprehensive knowledge on the relevance of the effect of the HBV genotype on serum pgRNA and HBcrAg levels.

## CONCLUSIONS

pgRNA and HBcrAg show diverse and corresponding profiles in different phases among pregnant CHB patients. Consistent with findings in nonpregnant patients with CHB, serum pgRNA and HBcrAg levels were lower in HBeAg-negative patients than in HBeAg-positive patients; serum pgRNA and HBcrAg levels correlated well with HBV DNA irrespective of HBeAg status but correlated significantly only with HBsAg among HBeAg-positive individuals.

## Supplementary Material

ofae241_Supplementary_Data

## Supplementary Data

Supplementary materials are available at *Open Forum Infectious Diseases* online. Consisting of data provided by the authors to benefit the reader, the posted materials are not copyedited and are the sole responsibility of the authors, so questions or comments should be addressed to the corresponding author.
