# Supplementary material for: Profiles of HBcrAg and pgRNA in Pregnant Women With Chronic HBV Under Different Disease Phases and Antiviral Prophylaxis
Source: Open Forum Infect Dis. 2024 May 3;11(5):ofae241. doi: 10.1093/ofid/ofae241 (PMC11097205; doi:10.1093/ofid/ofae241)
Supplement: ofae241_Supplementary_Data [file ofae241_supplementary_data.zip › Revised Table S1.docx]

| **Table S1 Clinical characteristics of 121 untreated patients.** | | | | | | |
| --- | --- | --- | --- | --- | --- | --- |
|  | HBeAg positive patients (N=100) | |  | HBeAg negative patients (N=21) | |  |
| **Parameter** | Immune tolerant phase (IT)  N=96 | Indeterminant phase (IND)  N=4 | *P1*  value | Immune active phase (IA)  N=6 | Inactive carrier phase (IC)  N=15 | *P2*  value |
| **Age, years** | 28.0 (26.0-31.0) | 25.5 (24.8-28.5) | 0.377 | 32.5(30.2-35.5) | 28.0(27.0-29.0) | 0.018 |
| **Parity status** |  |  | 0.222 |  |  | 0.281 |
| The first | 91 (94.8%) | 3 (75.0%) |  | 6 (100.0%) | 11 (73.3%) |  |
| The second | 5 (5.2%) | 1 (25.0%) |  | 0 (0.0%) | 4 (26.7%) |  |
| **Infant's gender, Male, n (%)** | 43 (44.8%) | 1 (25.0%) | 0.628 | 4 (66.7%) | 8 (53.3%) | 0.659 |
| **ALT, U/L** | 23.5(16.0-32.2) | 27.4(18.2-41.1) | 0.444 | 60.0(44.8-90.2) | 16.0(13.0-21.0) | <0.001 |
| **ALT categories, n (%)** |  |  | 0.526 |  |  | <0.001 |
| ≤19 U/L, n (%) | 39(40.63%) | 2(50.00%) |  | 0(0.00%) | 9(60.00%) |  |
| >19-40 U/L, n (%) | 47(48.96%) | 1(25.00%) |  | 1(16.67%) | 6(40.00%) |  |
| >40 U/L, n (%) | 10(10.42%) | 1(25.00%) |  | 5(83.33%) | 0(0.00%) |  |
| **Positive detection rate, n (%)**^a^ |  |  |  |  |  |  |
| PgRNA | 96(100.00%) | 2(50.00%) | <0.001 | 3(50.00%) | 4(26.67%) | 0.306 |
| HBV DNA | 96(100.00%) | 2(50.00%) | <0.001 | 6(100.00%) | 10(66.67%) | 0.105 |
| HBsAg | 96(100.00%) | 4(100.00%) | NA | 6(100.00%) | 15(100.00%) | NA |
| HBcrAg | 96(100.00%) | 4(100.00%) | NA | 6(100.00%) | 12(80.00%) | 0.237 |
| **PgRNA, log_10_copies/mL** | 7.8(7.6-8.1) | 2.2(0.0-4.6) | <0.001 | 2.3(0.0-5.6) | 0.0(0.0-1.2) | 0.211 |
| **HBV DNA, log_10_IU/mL** | 6.9(6.6-7.4) | 1.6(0.0-3.3) | <0.001 | 5.5(5.4-6.1) | 3.0(0.0-3.3) | <0.001 |
| **PgRNA/ HBV DNA ratio [log_10_ copies/mL/ IU/mL]** | 0.9 (0.4-1.2) | 0.4 (0.0-1.1) | 0.612 | -2.9 (-5.5--0.2) | -1.1 (-3.0-0.0) | 0.153 |
| **HBsAg, log_10_IU/mL** | 4.5 (4.3-4.7) | 3.2 (3.1-3.2) | 0.001 | 3.5(3.2-3.7) | 3.1(2.6-3.3) | 0.311 |
| **HBcrAg, log_10_U/mL** | 8.5(8.4-8.7) | 5.7(5.3-5.8) | <0.001 | 6.2(6.0-6.2) | 3.4(3.0-4.3) | 0.029 |
| **HBeAg, log_10_PEIU/mL** | 3.2 (2.9-3.3) | 0.4 (0.4-0.5) | <0.001 | NA | NA | NA |
| **APRI** | 0.3 (0.3-0.5) | 0.2 (0.2-0.3) | 0.067 | 0.5(0.3-1.8) | 0.3(0.2-0.3) | 0.043 |
| **APRI categories, n (%)** |  |  | 0.771 |  |  | 0.019 |
| ≤1, n (%) | 94(97.92%) | 4(100.00%) |  | 4(66.67%) | 15(100.00%) |  |
| >1, n (%) | 2(2.08%) | 0(0.00%) |  | 2(33.33%) | 0(0.00%) |  |
| **FIB-4** | 0.8 (0.6-1.0) | 0.4 (0.3-0.6) | 0.012 | 0.8(0.7-1.0) | 0.7(0.6-1.1) | 0.275 |
| **FIB-4 categories, n (%)** |  |  | 0.640 |  |  | 0.844 |
| ≤1.45, n (%) | 89(92.71%) | 4(100.00%) |  | 5(83.33%) | 13(86.67%) |  |
| >1.45, n (%) | 5(5.21%) | 0(0.00%) |  | 1(16.67%) | 2(13.33%) |  |

Abbreviations: Continuous variables were expressed as median [interquartile range (IQR)], and categorical variables were expressed as counts (percentage). MTCT, mother-to-child transmission; TDF, tenofovir disoproxil fumarate; LDT, telbivudine; HBV, hepatitis B virus; DNA, deoxyribonucleic acid; ALT, alanine aminotransferase; HBsAg, hepatitis B surface antigen; HBeAg, hepatitis B e antigen; pgRNA, pregenomic RNA; HBcrAg, hepatitis B core-related antigen. APRI, Aspartate aminotransferase-to-Platelet Ratio Index; FIB-4, Fibrosis 4 score. NA= not available.

^a^ Positive Detection Rate of pgRNA, HBV DNA, HBsAg and HBcrAg " would refer to the proportion of individuals in a group that test positive for the presence of pgRNA, HBV DNA, HBsAg and HBcrAg, respectively.
