# Supplementary figures and images for: Profiles of HBcrAg and pgRNA in Pregnant Women With Chronic HBV Under Different Disease Phases and Antiviral Prophylaxis
Source: Open Forum Infect Dis. 2024 May 3;11(5):ofae241. doi: 10.1093/ofid/ofae241 (PMC11097205; doi:10.1093/ofid/ofae241)

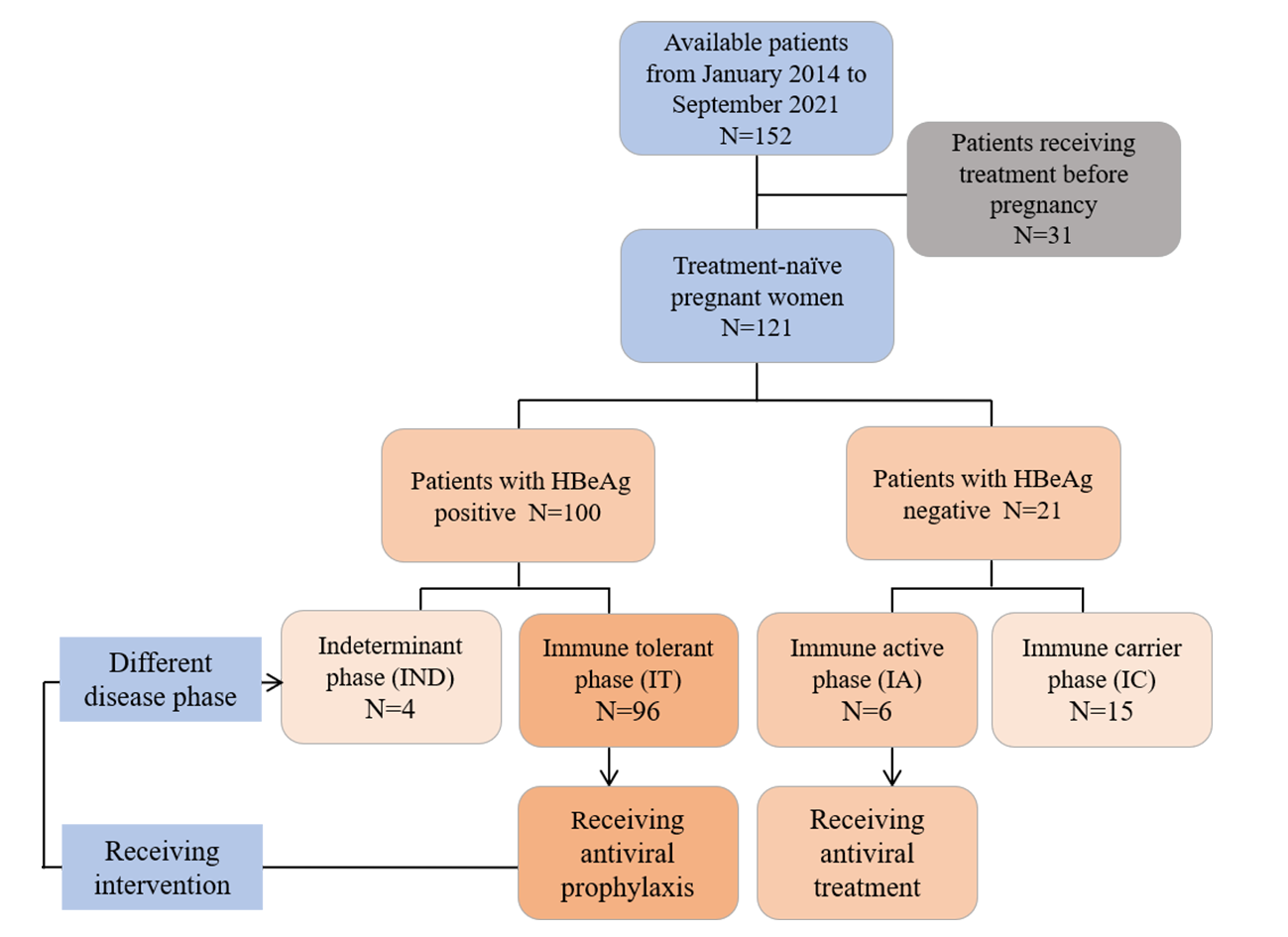

Supplement: ofae241_Supplementary_Data [file ofae241_supplementary_data.zip › Figure S1.tif]

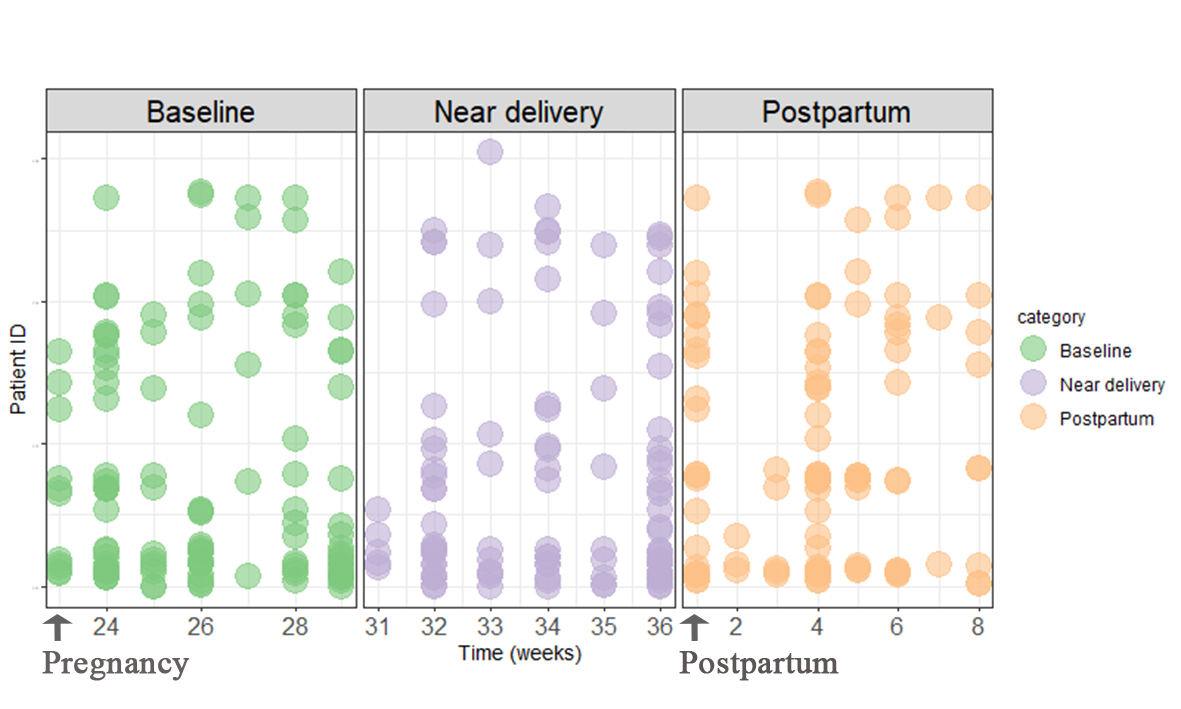

Supplement: ofae241_Supplementary_Data [file ofae241_supplementary_data.zip › Figure S2.tif]

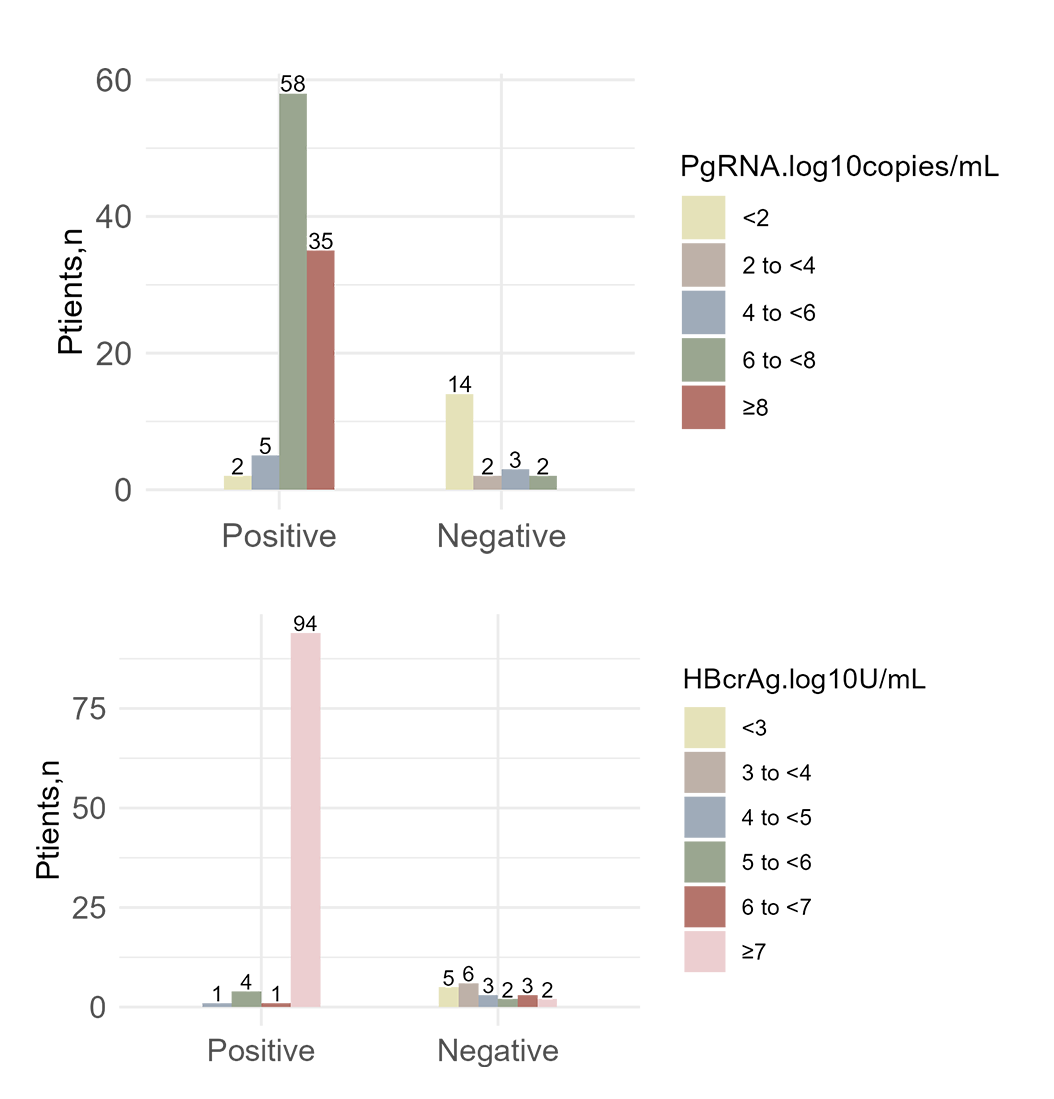

Supplement: ofae241_Supplementary_Data [file ofae241_supplementary_data.zip › Figure S3.tif]
